# Evaluation of the New Digital Goldmann Applanation Tonometer for Measuring Intraocular Pressure

**DOI:** 10.1155/2014/461681

**Published:** 2014-07-10

**Authors:** Yuta Sakaue, Jun Ueda, Masaaki Seki, Takayuki Tanaka, Tetsuya Togano, Takaiko Yoshino, Takeo Fukuchi

**Affiliations:** Division of Ophthalmology and Visual Science, Niigata University Graduate School of Medical and Dental Sciences, 1-757 Asahimachi-dori, Niigata 951-8510, Japan

## Abstract

*Purpose*. To compare a new digital Goldmann applanation tonometer (dGAT) that measures intraocular pressure (IOP) in 0.1 mmHg increments to a standard Goldmann applanation tonometer (sGAT). *Methods*. This study included 116 eyes of 60 subjects. A single examiner first measured IOP in triplicate using either sGAT or dGAT, which was randomly chosen. After a 5-minute interval, the next set of three consecutive IOP was measured using the other GAT. *Results*. The mean IOP measured with sGAT was 16.27 ± 6.68 mmHg and 16.35 ± 6.69 mmHg with dGAT. Pearson's correlation coefficient was 0.998 (*P* < 0.01). The subjects were divided into three groups based on the mean IOP: IOP < 14 mmHg, 14–20 mmHg, or >20 mmHg. The Pearson's correlation coefficient within each group was 0.935, 0.972, and 0.997 (*P* < 0.01), respectively. The difference within the three consecutive IOP measurements (maximum–minimum) for dGAT (0.72 ± 0.34 mmHg) was significantly smaller than those with sGAT (0.92 ± 0.42 mmHg, *P* < 0.01). Even in patients with equal IOP (zero left-right difference) with sGAT (*n* = 30), dGAT detected IOP differences between the left and right eyes (0.47 ± 0.31 mmHg). *Conclusion*. Compared to sGAT, dGAT measurements are highly reproducible and less variable.

## 1. Introduction

The pathogenesis and long-term natural history of glaucomatous optic neuropathy is still under active investigation. Many clinical trials have confirmed the key role intraocular pressure (IOP) plays in the development and progression of open-angle glaucoma [1–14]. Such studies have shown that lowering IOP reduces the risk of developing open-angle glaucoma and slows its progression. According to the Ocular Hypertension Treatment Study (OHTS) [1, 2], the risk of developing glaucoma is reduced from 9.5% to 4.4% when a mean IOP reduction of 22.5% was achieved with topical medications. Treatments that reduce IOP also reduced the proportion of patients with progression of clinically apparent glaucoma from 62% to 45% in the Early Manifest Glaucoma Trial (EMGT) [3–6], and from 27% to 12% in the Collaborative Normal-Tension Glaucoma Study (CNTGS) [7–9].

The current treatment strategy for glaucoma is to lower IOP in order to suppress the progression of glaucomatous optic neuropathy. Tonometry is one of the most important examinations in glaucoma management. With more accurate tonometry, more precise evaluation of reductions in IOP or the effects of glaucoma management may be possible.

The Goldmann applanation tonometer (GAT) is one of the most commonly accepted instruments to measure IOP. Despite the benefits of noncontact tonometers, they have a larger coefficient of variation than that of GAT and can result in larger measurement errors in patients with IOP < 10 mmHg or >25 mmHg [15]. The Tono-Pen (Medtronic Solan, Jacksonville, FL, USA) is more likely to have higher variability than GAT and produces lower readings in patients with ocular tension ≥20 mmHg [16]. GAT is widely adopted as the gold standard in tonometry due to its accuracy and excellent reproducibility. However, the standard Goldmann applanation tonometer (sGAT) has 2 mmHg markings on the drum, which can lead to disadvantages such as variability due to reading or digit preference [17, 18].

**Figure 1:**
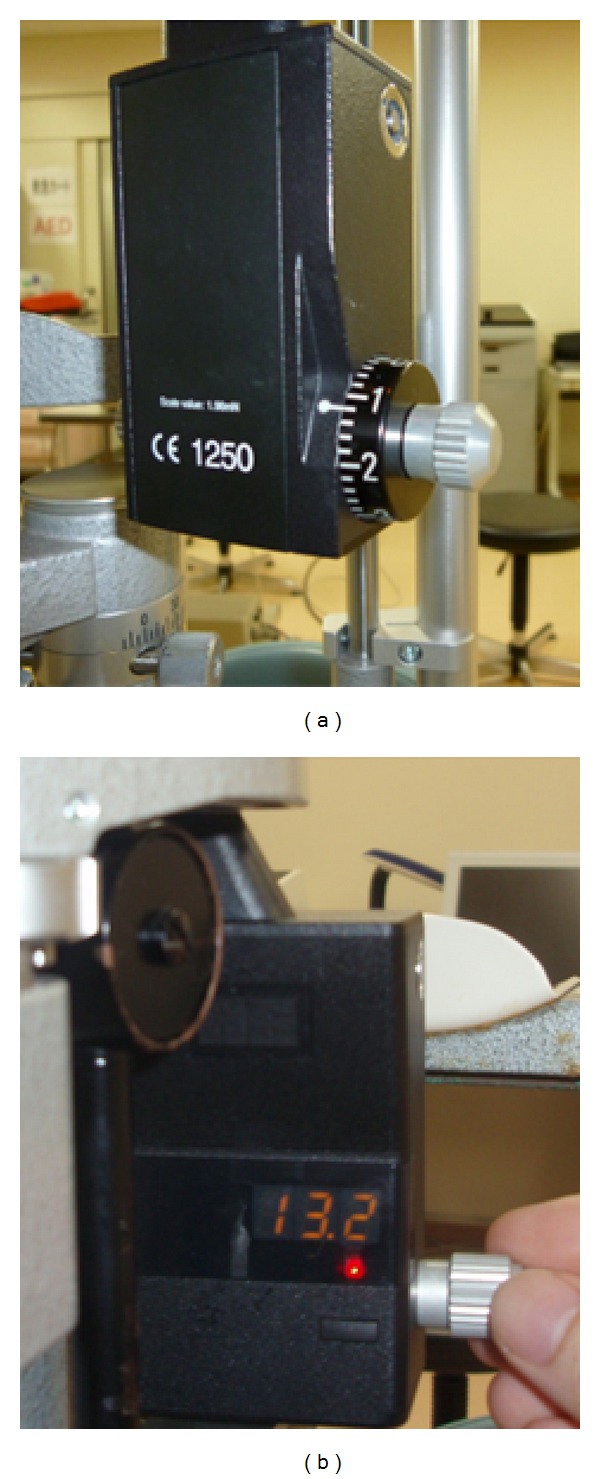
(a) The standard Goldmann applanation tonometer (sGAT: AT900, Haag-Streit International, Koeniz, Switzerland); and (b) the digital Goldmann applanation tonometer (dGAT: AT900D, Haag-Streit International).

AT900D (Haag-Streit International, Koeniz, Switzerland) is a new digital Goldmann applanation tonometer (dGAT). The principles and basic methods for IOP measurement are the same as those of sGAT; however, the measurements are shown to the 0.1 mmHg level on the display (Figure 1).

In this study, measurements obtained with sGAT and dGAT were compared to assess the accuracy and possible advantages of dGAT.

## 2. Materials and Methods

This study included 116 eyes of 60 subjects, including 15 eyes of 8 healthy subjects and 101 eyes of 52 patients with glaucoma.  Table 1 shows the profiles of the participants. Patients with a history of surgery for glaucoma and corneal disease were excluded. The study protocol was designed according to the norms of the Declaration of Helsinki. Written informed consent was obtained from the participants.

The sGAT employed in this study was the AT900 (Haag-Streit International). Although the instrument has 2 mmHg markings on the drum (Figure 1), measurements were made to the 1 mmHg level. The dGAT employed in this study was the AT900D. Digital measurements were obtained to the 0.1 mmHg level (Figure 1). For IOP measurement, the eye was anesthetized with 0.4% oxybuprocaine. Fluorescein was applied to the inferior fornix using a standard fluorescein paper strip. A single examiner (Y.S.) first measured IOP in triplicate for each eye using either sGAT or dGAT, which was randomly chosen. After a 5-minute interval, the next set of three consecutive IOP measurements was obtained with the other GAT. The three IOP readings were averaged to obtain IOP for the eye.

The mean IOP in the right eye of the 60 patients as determined by sGAT and dGAT were compared using Pearson's correlation analysis. A Bland-Altman plot was constructed to evaluate agreement and calculate confidence intervals (CIs). For each eye, differences in IOP measurements (maximum–minimum) based on sGAT and dGAT were compared to study the dispersion in measurements using Student's *t*-test. A *P* value <0.05 was considered to indicate statistical significance. The second of the three sGAT and dGAT readings for each eye was used to evaluate differences between IOP in the left and right eye. The left-right difference in IOP measured by dGAT was calculated in patients with equal left and right sGAT measurements.

## 3. Results

**Figure 2:**
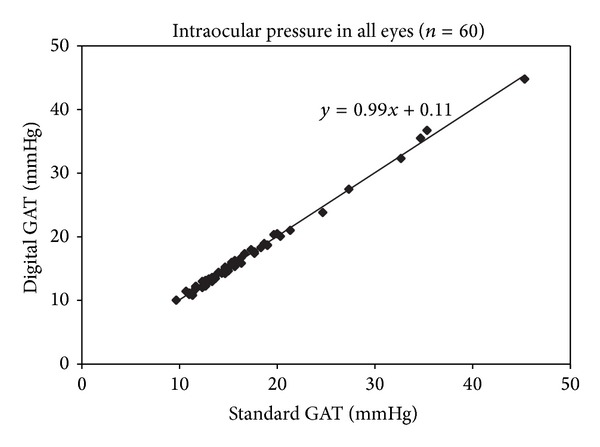
Scatter plot of intraocular pressure as measured by standard Goldmann applanation tonometry (GAT) and digital GAT. Standard and digital GAT measurements of intraocular pressure were positively correlated (*r* = 0.998, *P* < 0.01, Pearson's correlation analysis).

**Figure 3:**
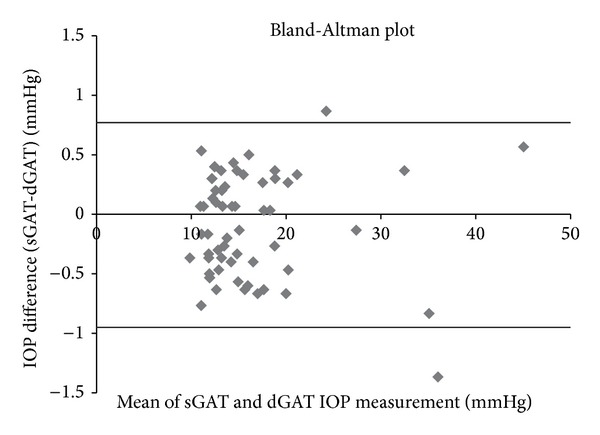
Bland-Altman plot of mean intraocular pressure (IOP) based on standard Goldmann applanation tonometry (sGAT) and digital Goldmann applanation tonometry (dGAT). The mean difference (sGAT-dGAT) was –0.09 mmHg. The 95% confidence interval (mean difference ± 1.96 SD) ranged from –0.95 mmHg to +0.77 mmHg (*n* = 60) as shown by broad lines in the graph.

The mean IOP of the 60 eyes measured using sGAT was 16.27 ± 6.68 mmHg and that for dGAT was 16.35 ± 6.69 mmHg. Pearson's correlation analysis revealed a significant positive correlation between sGAT and dGAT measurements of IOP (Table 2 and Figure 2: *r* = 0.998, *P* < 0.01). When the subjects were divided into three groups based on the mean sGAT IOP value (≤14 mmHg, 30 eyes; 14–20 mmHg, 21 eyes; or ≥20 mmHg, 9 eyes), sGAT and dGAT measurements within each stratum showed strong positive correlation (Table 2: *r* = 0.935, *P* < 0.01; *r* = 0.972, *P* < 0.01; and *r* = 0.997, *P* < 0.01; resp.). According to Bland-Altman analysis, dGAT measurements showed no skew compared to sGAT measurements (Figure 3, *n* = 60). The mean difference (sGAT-dGAT) in IOP readings for each eye was −0.09 ± 0.44 mmHg (*n* = 60). The difference (maximum–minimum) within each set of three dGAT measurements was 0.72 ± 0.34 mmHg, which was significantly smaller than that for sGAT (0.92 ± 0.42 mmHg, *P* < 0.01, *n* = 60).

The second reading in each set of sGAT or dGAT measurements was used to analyze differences between left and right eyes, because it was closest to the mean of the three tonometry measurements. Of 60 patients examined, 30 patients had equal IOP in both eyes based on sGAT (i.e., zero left-right difference). The mean left-right difference detected by dGAT in these 30 patients with no left-right difference on sGAT was 0.47 ± 0.31 mmHg (range: 0-1.0 mmHg).

## 4. Discussion

New tonometers such as the rebound tonometer and dynamic contour tonometer have been developed recently. The rebound tonometer is portable and does not require topical anesthesia. However, it is likely to show higher IOP measurements than those of GAT [19, 20]. The dynamic contour tonometer is less influenced by central corneal thickness and is also more likely to show higher IOPs than GAT [21, 22]. Therefore, while further studies have been investigating the characteristics of these tonometers, GAT is still considered as the gold standard because of its clinically proven accuracy and availability.

Measurements with GAT are usually made to the 1 mmHg level with a sGAT's scale marked at every 2 mmHg. If a measurement lies between the lines on the scale, the measurement is subjectively judged by the examiner. Thus, the results may differ between examiners. IOP measurements by dGAT are displayed to the 0.1 mmHg level, resulting in more objective measurements. Another benefit of dGAT is that IOP data on the display can be easily read in a dark room (Figure 1).

Previous studies have reported that dGAT yields highly reproducible results and there is a significant positive correlation between sGAT and dGAT measurements [23, 24]. Similarly, in the present study, we observed significant positive correlation between sGAT and dGAT measurements (Figure 2 and Table 2). Furthermore, we observed a positive correlation between sGAT and dGAT results even when the subjects were divided into groups based on the mean tonometry reading (Table 2). Before the present study, the dispersion in measurements based on differences (maximum–minimum) has not been investigated. This study demonstrated that dGAT yields significantly smaller differences (maximum–minimum) when sets of three consecutive tonometry readings are analyzed. The amount of dispersion was smaller presumably due to the 0.1 mmHg resolution. We also found that there was no trend for skew in the sGAT and dGAT readings based on the Bland-Altman plot (Figure 3). These results may be a reflection of the fact that sGAT and dGAT are based on the same principles to measure IOP.

Even in patients with equal IOP (zero left-right difference) with sGAT, dGAT detected IOP differences between the left and right eyes. Although the differences were small (0.47 ± 0.31 mmHg), this result indicates that even small differences in IOP that could not be detected by sGAT can be detected by dGAT. This characteristic of dGAT may be useful when evaluating the effects of medications, especially in patients with normal-tension glaucoma or those who achieved low IOP with treatment. In such patients, changes in IOP too small to be detected by the 1 mmHg resolution with sGAT can be observed with dGAT with 0.1 mmHg resolution.

In conclusion, dGAT, which shares the same principles for IOP measurement with sGAT, can provide more accurate IOP data with high reproducibility and less dispersion due to its 0.1 mmHg scale. Thus, dGAT will enable the more refined IOP evaluation required for clinical management of patients with normal-tension glaucoma and patients with progressive visual field loss despite low IOP.

## Figures and Tables

**Table 1:** Profiles of the study subjects (116 eyes of 60 patients).

Sex	
Male	61 eyes of 32 patients
Female	55 eyes of 28 patients
Age (mean ± SD)	57.6 ± 19.0 years
Diagnosis	eyes
Primary open-angle glaucoma	38
Normal-tension glaucoma	37
Developmental glaucoma	8
Exfoliation glaucoma	5
Chronic angle-closure glaucoma	6
Uveitis and secondary glaucoma	7
Healthy	15

**Table 2:** Mean intraocular pressure readings in the study subjects.

	All eyes	Group 1	Group 2	Group 3
		(IOP ≤ 14)	(14 < IOP < 20)	(IOP ≥ 20)
Number of patients	60	30	21	9
Standard GAT (mmHg)	16.27 ± 6.68	12.26 ± 1.01	16.49 ± 1.72	29.07 ± 8.58
Digital GAT (mmHg)	16.35 ± 6.69	12.37 ± 0.97	16.59 ± 1.78	29.11 ± 8.71
Pearson's correlation coefficient	0.998	0.935	0.972	0.997
*P* value	<0.01	<0.01	<0.01	<0.01

IOP: intraocular pressure; GAT: Goldmann applanation tonometer.

Pearson's correlation coefficient test.
